# Effect of (R)-(+) Pulegone on Ovarian Tissue; Correlation with Expression of Aromatase Cyp19 and Ovarian Selected Genes in Mice

**DOI:** 10.22074/cellj.2018.4798

**Published:** 2018-03-18

**Authors:** Rohiyeh Souldouzi, Mazdak Razi, Ali Shalizar Jalali, Ghader Jalilzadeh-Amin, Saeedeh Amani

**Affiliations:** 1 Department of Basic Sciences, Faculty of Veterinary Medicine, Urmia University, Urmia, Iran; 2 Department of Veterinary Internal Medicine, Faculty of Veterinary Medicine, Urmia University, Urmia, Iran

**Keywords:** Apoptosis, Aromatization, Mice, Ovary, Pulegone

## Abstract

**Objective:**

Pulegone (PGN) is a monoterpene ketone, whose metabolites exert several cytotoxic effects in various tissues. The present study was conducted in order to evaluate the (R)-(+) PGN-induced alterations in ovarian aromatization, proto-oncogenes and estrogen receptorα (ERα) and ERβ receptors expressions.

**Materials and Methods:**

In this experimental study, mature albino mice were divided into experimental (received 25 mg/kg, 50 mg/kg and 100 mg/kg PGN, orally for 35 days) and control (received 2% solution of Tween 80 as a PGN solvent, orally) groups. The mRNA levels of *Erα, Erβ, p53, Bcl-2,* and *cytochrome p450 (Cyp19)* as well as ovarian angiogenesis were analyzed through reverse transcription polymerase chain reaction and immunohistochemical techniques, respectively. Moreover, apoptosis of follicular cells, serum estrogen and progesterone levels and mRNA damage were investigated via using terminal transferase and biotin-16-dUTP staining, electrochemilunescence and fluorescent microscopy methods, respectively.

**Results:**

The PGN reduced *Erα, Erβ* and *Cyp19* expression at 50 mg/kg and 100 mg/kg doses, while significantly elevating *p53* and reducing *Bcl-2* expression. Finally, PGN impaired ovarian angiogenesis, increased apoptosis, elevated follicular atresia and reduced serum levels of estrogen and progesterone.

**Conclusion:**

Chronic exposure to PGN (50 mg/kg and 100 mg/kg), severely affects ovarian aromatization, proto- oncogenes mRNA levels and expression of ERs.

## Introduction

Pulegone (PGN), a monoterpene ketone, is a significant constituent of several mint (Mentha) species and their derived volatile oils, including peppermint (*Mentha piperita*), spearmint (*Mentha spicata*), European pennyroyal (*Mentha pulegium L.*) and American pennyroyal (*Hedeoma pulegioides L.*) (1). The pennyroyal leaves are used to prepare tea, which has been recommended as an aromatic stimulant, carminative, emmenagogue, and a headache remedy.Thus, the induced impacts are unpredictable and dangerous. Beside high consumption through different varieties of pennyroyals, low levels of pure PGN are used for flavoring foods, drinks, and dental products (2). 

It should be noted that intake of PGN also leads to exposure to menthofuran, which is a major metabolite of PGN in the body (3). According to several reports, PGN and its metabolites such as piperitenone, piperitone, menthofuran, and menthone have several cytotoxic impacts on various tissues (2, 4). No treatment-mortality was observe d in male or female rats which received 0, 9.375, 18.75, 37.5, 75, or 150 mg of PGN/kg body weight in corn oil by gavage, 5 days per week for 14 weeks. However, the two highest doses (75 and 150 mg/ kg) caused several adverse effects including, weight loss, increased absolute and relative liver and kidney weights, hyaline glomerulopathy, bile duct hyperplasia and hepatocyte hypertrophy (5).

In another study, PGN administration at the doses of 0.75 mg/kg and 150 mg/kg resulted in superficial necrosis of the bladder epithelium and exfoliation (6). In line with this issue, several cases of pennyroyal toxicity have been reported (7, 8). Most cases have occurred in adult women who used pennyroyal as an abortifacient and some of these cases have even resulted in death (2). The ability of liver cytochromes (CYPs) to catalyze PGN oxidations have been examined previously. It has been shown that CYP2E1, CYP1A2, and CYP2C19 are able to oxidize PGN to menthofuran (2). Indeed, in several tissues such as the ovary, placenta, brain and testis the estrogen receptors (*Ers*) are involved/co-expressed in encoding the enzyme aromatase p450 (9-11). The CYP enzymes directly aromatize the androgens to estrogen, which in turn plays essential roles in follicular growth and various ovarian physiological functions (12, 13).

The effects of estrogen are mediated by two distinct estrogen receptors, *Er-α*(14) and *Er-ß* (15, 16) that both regulate expression of a variety of different genes. Any disruption in the expression of these genes affects the estrogen signaling system, leading to reduced proliferation and differentiation in both male and female gonads (16, 17). Correlating with the enclosed interactions of *CYPs* and Ers, it should be noted that ERα knockout (*αERKO*) leads to severe ovarian hemorrhage cyst generation, failure of follicular growth and maturation as well as ovulation (14, 15). However, the *ßERKO* mice exhibit grossly normal ovarian tissue with follicles at different stages of growth/development but fewer corpora lutea (18, 19). 

Lastly, it has been shown that, there is a link between groups of genes including, ERs and progesterone receptors (PRs) with *Bcl-2* and *p53*, which significantly affects the apoptosis and/or proliferation ratio (20). Indeed, *Bcl-2* and *p53* are considered as genes that are responsible for the initiation, progression and completion of apoptosis. Accordingly, *Bcl-2* promotes cell survival by inhibiting protease activation and is known as a key regulator of apoptosis at early stages (21, 22). The *p53* has been dubbed the guardian of the cell’s genome as it stabilizes and accumulates in the nucleus of cells with DNA damage that are undergoing replication. Therefore, *p53* is both positively or negatively associated with apoptosis (23, 24).

Considering the role of aromatase enzymes in oxidizing PGN, the present study was designed to evaluate the probable effect of PGN on ovarian *Cyp19* (as a main enzyme involved in PGN metabolism). Moreover, we aimed to analyze the effect of chronic exposure to PGN on ovarian histological features. Ultimately, in order to illustrate the possible roles of genes involved in follicular atresia, the mRNA levels and immunohistochemical measurements of the co-associated genes such as *Bcl-2, p53, Erα* and *Erß* were investigated.

## Materials and Methods

In this experimental study, PGN was bought from Sigma Co. (CAS NO: 89-82-7). The acridine-orange was purchased from sigma chemical Co. (St. Louis, MO, USA). Tween 80 was obtained from Merk (Germany). The rabbit anti- mouse primary antibodies for *Erα* and *Erß* (Biocare, USA) as well as CD31 (Gennova, Spain) were assigned from Pishtaz Teb Co. (Iran). The 3,3’-Diaminobenzidine (DAB) chromogen was from Agilent technologies Co. (DAKO, Turkey). Mounting medium for immunohistochemical analysis (VECTASHIELD) was from Vector Laboratories (Burlingame, CA, USA). Other used materials were standard commercial laboratory chemicals.

### Animals and expermintal groups

For this study, we used 40 mature (average of 10 weeks old) albino mice (Urmia University, Iran) with high heterozygosity and average weight of 20-25 g. Mice were divided into experimental and control groups (10 mice for each group) and kept under standard experimental conditions (constant temperature and 12-hour light regime). Animals were fed soy-free feed. The diet and water were administered ad libitum and all stress factors were reduced to a minimum.

Experimental groups were treated with different concentrations of PGN, which was administrated orally by gavage. The experimental group was divided into 3 subgroups: a- received 25 mg/kg PGN, b- received 50 mg/ kg PGN and c- received 100 mg/kg PGN. The animals in the control group received 2% solution of Tween 80 as the solvent for PGN (25). The animals received PGN and Tween 80 for 35 continuous days. All necessary ethics were considered during the study and the procedures were approved by the Ethical Committee of Urmia University (number AECVU/136/2016).

### Histological analyses 

After 35 days, the ovaries were dissected and fixed in 10% formalin for 72 hours. Then, the ovaries were seperated from per-ovarian tissues under high magnification using a stereo microscope (Olympus, Japan). The routine sample processing was performed for the right and left ovaries (5 ovaries from each side, total 10 ovaries from 5 mice of each experimental group) and samples were embedded in paraffin blocks which were serially cut using a rotary microtome and stained with hematoxylin-eosin. For histomorphometric analyses, follicles were classified into preantral (<100 and 100-200 µm) and antral (201-400 µm).

Follicular morphology was examined under light microscope with ×200 magnification. Follicles with an intact layer of normal granulosa and flattened theca cells, oocytes with ordinary cytoplasm and nuclei were considered as normal follicles. Follicles were classified as abnormal if we witnessed granulosa cells (GCs) dissociation, early antrum formation, GCs luteinization and floatation in antrum. Follicular count was estimated by counting follicles in all serially prepared slides. Moreover, the atretic preantral and antral follicles were counted in serial sections for each sample and compared between groups (26).

### Fluorescent assessment of RNA damage

The RNA damage was assessed based on the Darzynkiewicz method (27). In brief, the ovaries were washed out with ether alcohol and cut using a cryostat (8 µm). The prepared sections were then fixed by different concentrations of ethanol for 15 minutes. The sections were rinsed in acetic acid (1%) and then washed in distilled water. The specimens were stained in acridineorange for 3-5 minutes and counterstained in phosphate buffer (pH=6.85). After that, the slides were checked for change in fluorescent colors in calcium chloride. The loss of RNA in necrotic follicular cells was characterized by faint red stained RNA. The normal cells were marked bright red at the apex of the nucleus.

### Apoptosis detection using terminal transferase and biotin-16-dUTP

In order to analyze programmed death of single cells, the terminal transferase and biotin-16-dUTP staining technique was used. In brief; the sections (6 µm) were immersed twice in xylene for 5 minutes and then 2 immersions were performed for hydration in 100% ethanol for 3 minutes and then the slides were rinsed in distilled water. The slides then were digested in Proteinase K [10 mg/ml stock in 100 mM Tris-HCl (pH=7.5), 10 mM EDTA] and rinsed in PBS (pH=7) 2 times, 3 minutes each. The slides were pre-incubated in TdT reaction buffer (Enzyme reagent 100 µl, label reagent 900 µl) for 10 minutes. Following pre-incubation, the slides were incubated in TdT reaction mixture for 1-2 hours at 37°C in a humidified chamber.

To stop the reaction stage, the slides were rinsed in stop reaction buffer (NaCl 1.75 g, Sodium citrate, Trihydrate 0.88 g, distilled water 100 ml) for 10 minutes. The sections were incubated in FITC-Avidin D in phosphate-buffered saline (PBS) for 30 minutes at room temperature. After 3 immersions in PBS, the slides were counterstained in PI. Then, the slides were rinsed in PBS and mounted with anti- fading mounting medium (28). All slides were analyzed using a fluorescent microscope (Zeiss, Germany).

### Immunohistochemical assessment of angiogenesis 

Tissue sections were heated at 60°C for approximately 25 minutes in a hot air oven (Venticell, MMM, Einrichtungen, Germany). The tissue sections were de-paraffinized in xylene and rehydrated using an alcohol gradient (96, 90, 80, 70, 50%). A 10 mM sodium citrate buffer was used for the antigen retrieval process. Then, the IHC staining was conducted according to the manufacturer’s protocol (Biocare, USA). Briefly; endogenous peroxidase was blocked in a peroxidase blocking solution (0.03% hydrogen peroxide containing sodium azide) for 5 minutes.

Tissue sections were then washed gently with washing buffer and subsequently incubated with CD31 (1:500) biotinylated primary antibodies for 15 minutes. The sections were rinsed gently with washing buffer. The slides were then incubated in a humidified chamber with a sufficient amount of streptavidin-horseradish peroxidase (HRP) (streptavidin conjugated to horseradish peroxidase in PBS containing an anti-microbial agent) for 15 minutes. Subsequently, the tissue sections were rinsed gently in washing buffer and placed in a buffer bath. Then the slides were incubated with DAB chromogen for 5 minutes, followed by washing and counter staining with hematoxylin for 5 seconds.

The sections were then dipped in weak ammonia (0.037 M) 10 times, rinsed with distilled water and covered with cover slips. Positive immunohistochemical staining was observed as brown stains under a light microscope (×100 and ×400 magnification). The vessels were classified into 1-5 µm and 5-10 µm as newly generated vessels and previously existing vessels. The vascular distribution per mm^2^ of the ovarian tissue was compared between groups.

### Immunohistochemical analyses for ERα and ERß

The same protocol as IHC staining of the CD31 was performed for IHC of the ERα and ERß. Meanwhile the specific primary antibodies were used for each protein. The positive-stained cells were counted per 100 cells and compared between groups.

### RNA isolation

Total RNA was extracted from ovaries of experimental and control animals. For this extraction we used a Sina Clon RNA extraction kit (SinaGen, Iran). To each ovarian sample, 1 ml of Tris reagent was added and the tissue was then homogenized in a Precellys 24 homogenizer (Bertin Technologies, Aix-en-Provance, France). Subsequently, the samples were processed according to the manufacturer’s instructions. Isolated RNA was stored at -70°C. The RNA quality and purity were measured with a NanoDrop-1000 spectrophotometer (Thermo Scientific, Washington, USA).

### Reverse transcription polymerase chain reaction

Using reverse transcription polymerase chain reaction (RT-PCR), cDNA was synthesized in a 20 µl reaction mixture containing 1 µg RNA, oligo (dT) primers (1 µl) 5X reaction buffer (4 µl), RNAse inhibitor (1 µl), 10 mM dNTP mix (2 µl) and MMuLV reverse transcriptase (1 µl) according to the manufacturer’s protocol (Fermentas, GmbH, Germany). The cycling protocol for 20 µl reaction mixture was 5 minutes at 65°C followed by 60 minutes at 42°C, and 5 minutes at 70°C to terminate the reaction.

The obtained cDNA was stored at -20°C. PCR conditions were run as follows: general denaturation at 95°C for 3 minutes, followed by 40 cycles of 95°C for 20 seconds; annealing temperature (62°C for *Erα,* 58°C for *Erß,* 54°C for *p53,* 58°C for *Bcl-2,* 55°C for *Cyp19* and 63°C for *Gapdh*) for 45 seconds; elongation at 72°C for 1 minute and a final 72°C for 5 minutes. Specific primers were designed and manufactured by CinnaGen (Iran). The sequences, products size and annealing temperature for each of the primer pairs is depicted in Table 1. Final PCR products wereanalyzed on 1.5% agarose gel electrophoresis and the densitometric analysis of the bands was done by using PCR Gel analyzing software (ATP, Iran). The control was set at 100% and experimental samples were compared to the control. 

**Table 1:** Sequences of the primer pairs used


Gene (bp)	Primer sequencing (5ˊ-3ˊ)	Product size

*Erα*	F: TACGAAGTGGGCATGATGAA	194
	R: AAGGACAAGGCAGGGCTATT	
*Erβ*	F: CAAGAGAACCAGTGGGCACAC	273
	R: CAGCCAATCATGTGCACCAG	
*p53*	F: GGG ACA GCC AAG TCT GTT ATG	350
	R: GGA GTC TTC CAG TGT GAT GAT	
*Bcl-2*	F: GTGAACTGGGGGAGGATTGT	240
	R: GGAGAAATCAAACAGAGGCC	
*Cyp19*	F: CACCCTTCCAAGTGACAGGA	289
	R: AAAAAAGTAAAGTTCTATGGGAA	
*Gapdh *	F: GTTACCAGGGCTGCCTTCTC	310
	R: GGGTTTCCCGTTGATGACC	


### Blood sampling and hormonal analyses

The blood samples from corresponding animals were collected directly from the heart and the serum was separated by centrifugation (3000 g for 5 minutes) and subjected to assessment for serum progesterone and estrogen concentrations. Progesterone and estrogen were measured with the electrochemilunescence method. The intra-assay coefficient variance for estradiol and progesterone were, 5.9% (for 10 measurements) and 4.8% (for 10 measurements), respectively. Inter-assay coefficients variances of 8.9% (for 10 measurements) and 9.9% (for 10 measurements) were also calculated for estrogen and progesterone, respectively.

### Statistical analyses

All results are presented as mean ± SD. Differences between quantitative histological and hematological data were analyzed with two-way ANOVA, followed by Bonferroni test, using Graph Pad Prism 4.00 and P<0.05 was considered as statistically significant.

## Results

### Pulegone increased follicular atresia

**Fig.1:**
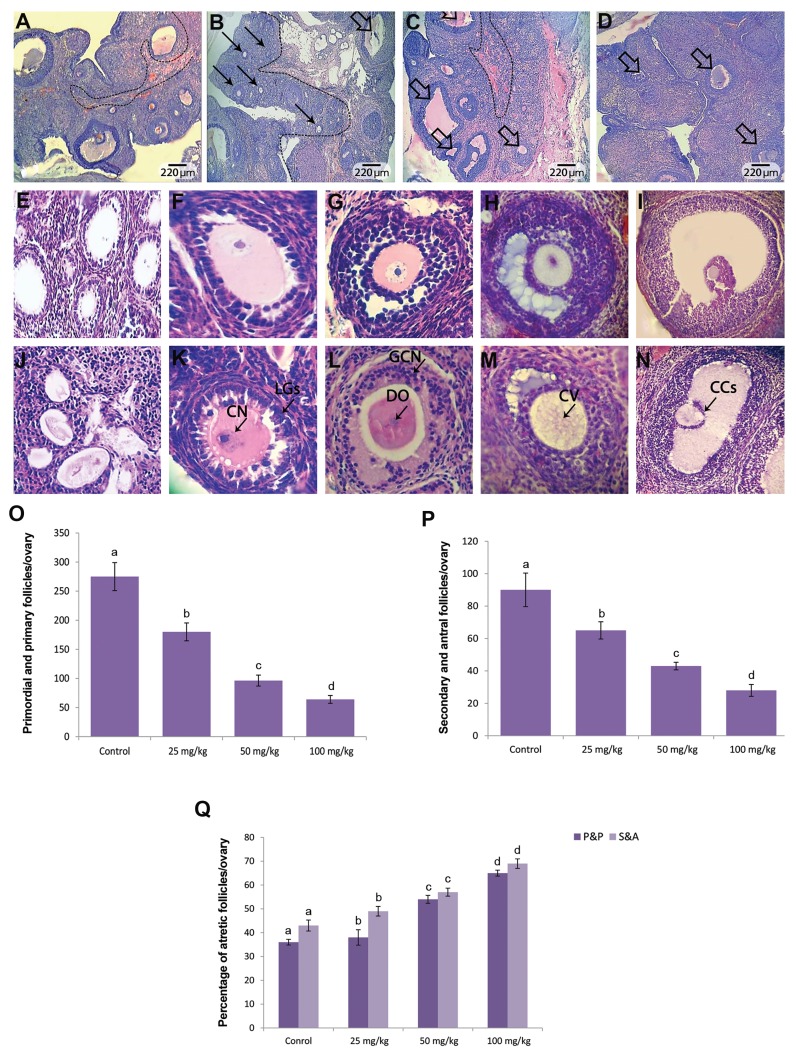
Cross section from ovaries. A. Control, B. Low dose pulegone (PGN)-exposed (25 mg/kg), C. Medium dose PGN-exposed (50 mg/kg), D. High dose PGN-exposed groups. Significantly higher antral follicles distribution and active ovary were seen in the control group. PGN reduced the follicular growth in a dose dependent manner. Note the small size (arrows) and antral (thick arrows) atretic follicles in the cortex region of PGN-exposed groups, E. Normal primary, F. Early secondary, G. Late secondary, H. Tertiary, I. Graafian follicles are presented in figures, J. Missing oocyte in atretic primary follicle, K. Centrifugal nuclei (CN) and luteinized granulosa cells (LGs) in atretic early secondary follicle, L. Disappeared oocyte (DO) associated with necrosis of granulosa cells (GCN) in atretic late secondary follicle, M. Cytoplasmic vacoulation (CV) of oocyte in atretic tertiary follicle, N. Cumulus cells abnormality (CCs) in atretic graafian follicle (H&E staining, ×200 and ×400 magnifications), O. Mean changes of primordial and primary, P. Secondary and antral follicles total count per ovary, and Q. Percentage of atretic primordial and primary (P&P), and secondary and antral (S&A) follicles per ovary in different groups. All data are presented as mean ± SD. Different letters represent significant (P<0.05) differences between marked groups.

Histological analyses showed that PGN, in a dose dependent manner, enhanced follicular atresia at both preantral and antral stages. Accordingly, the animals given high doses (100 mg/kg) of PGN demonstrated significantly (P<0.05) higher percentage of atretic follicles versus the medium dose (50 mg/kg) and low dose (25 mg/ kg) groups. The PGN-exposed ovaries exhibited pie size (<100 µm) atretic follicles in the cortex. The oocytes of the preantral follicles were found without nuclei and a faint eosinophilic and vacuolated cytoplasm. The GCs of the atretic preantral follicles were observed to have enlongated shapes. Antral atretic follicles appeared to be void of an oocyte and showed dissociated granulosa cells, increased thickness of the zona pelucida and noncontinuous cumulus oophorus (Fig .1A-Q). 

### Pulegone induced necrosis and apoptosis

**Fig.2:**
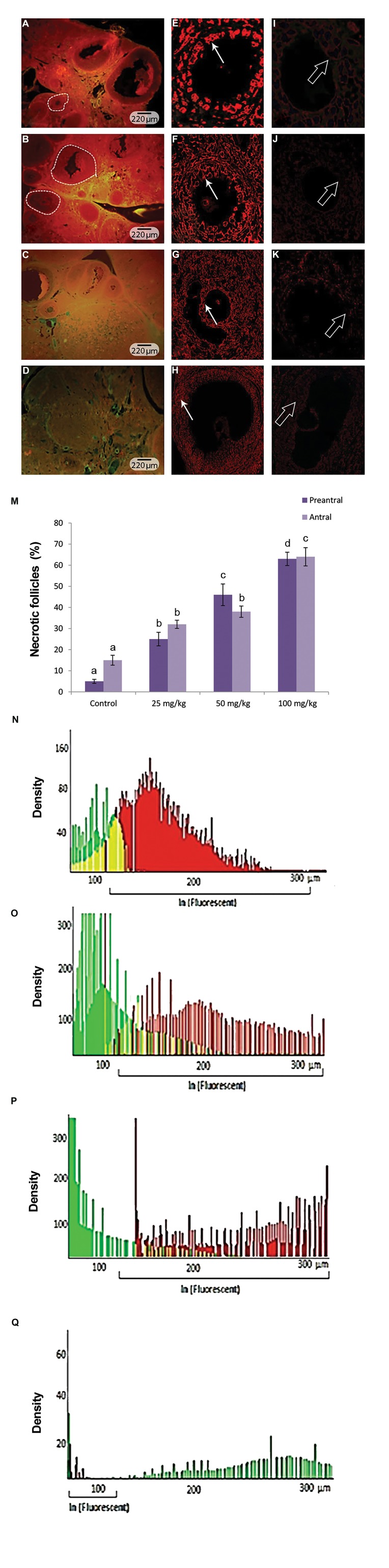
Fluorescent staining for mRNA damage. A. Control, B. Low dose pulegone (PGN)-exposed (25 mg/kg), C. Medium dose PGN- exposed (50 mg/kg), D. High dose PGN-exposed groups. Cross sections from PGN-exposed groups show dose dependent enhancementin the distribution of ovarian and follicular necrotic cells, E. Normal cells with intact mRNA content (red stained and marked with arrows) in intact primary, F. Secondary, G. Tertiary, H. Graafian follicles. However, the follicles in the right hand column are demonstrate mRNA damage in atretic follicles, exhibiting necrotic cells with faintstained yellowish red RNA in I. Primary, J. Secondary, K. Tertiary, L. Graafian follicles (Thick arrows, ×200 and ×400 magnifications), M. Mean percentage of follicles with necrotic cells in different groups. Alldata are presented as mean ± SD. ^a, b, c, d^ represent significant differences(P<0.01) between marked groups. Necrotic cells distribution per 300µm of the ovarian stroma in N. Control, O. Low dose PGN-exposed (25 mg/kg), P. Medium dose PGN-exposed (50 mg/kg), and Q. highdose PGN-exposed groups. Red bars represent normal cells with intactmRNA, green bars present cellular DNA content and yellow bars mark the connective tissue content of the ovaries. Decreased normal cells with intact mRNA were seen in PGN-exposed groups.

In order to evaluate the necrotic follicular and stromal cells, a special fluorescent staining for mRNA damage was performed. Observations demonstrated that, PGN (remarkably at dose of 100 mg/kg) increased mRNA damage and elevated the necrotic cells distribution. The software analyses for the necrotic cells (cells with yellowish RNA content) and the intact cells distribution (cell with dense red stained RNA content) showed a significant elevation in the distribution necrotic cells in 300 µm of the PGN-exposed ovarian stroma. Comparing the percentage of follicles with necrotic cells between test and control groups showed a remarkable elevation in the high dose (100 mg/kg) group. This impairment was revealed significantly (P<0.05) in lower in low (25 mg/kg) and medium (50 mg/kg) PGN-receiving groups (Fig .2A-Q).

**Fig.3:**
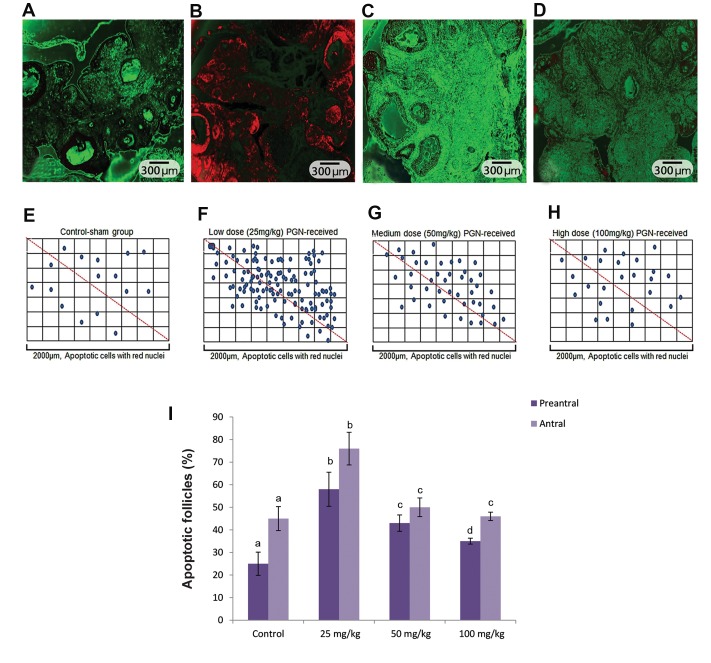
Apoptosis detection using terminal transferase and biotin-16-dUTP. A. Control, B. Low dose pulegone (PGN)-exposed (25 mg/kg), C. Medium dose PGN-exposed (50 mg/kg), D. high dose PGN-exposed groups. The apoptotic cells are discernable through their red stained nuclei. Intensive cellular apoptosis was seen in the low dose (25 mg/kg) PGN-exposed group, which is significantly decreased in the medium dose (50 mg/kg) and high dose (100 mg/kg) PGN-exposed groups (×200 magnification). Distribution of apoptotic cells per 2000 µm of ovarian tissue in E. Control, F. Low dose PGN-exposed (25 mg/kg), G. Medium dose PGN-exposed (50 mg/kg) and H. High dose PGN-exposed groups. Increased blue spots for apoptotic cells can be seen in the low dose (25 mg/kg) PGN-exposed group versus the medium dose (50 mg/kg) and high dose (100 mg/kg) PGN-exposed groups, I. Mean percentage of follicles with apoptotic cells in different groups. All data are presented as mean ± SD. ^a, b, c, d^ represent significant differences (P<0.01) between marked groups.

The fluorescent staining for apoptosis was performed as another possible mechanism for follicular atresia (especially in lower doses). Observations revealed that PGN at low (25 mg/kg) and medium (50 mg/kg) doses causes increased apoptosis at both follicular and stromal cells (Fig .3A-D). The Percentage of follicles with apoptotic cells are presented in Figure 3E-I.

### Pulegone affected ovarian angiogenesis 

**Fig.4:**
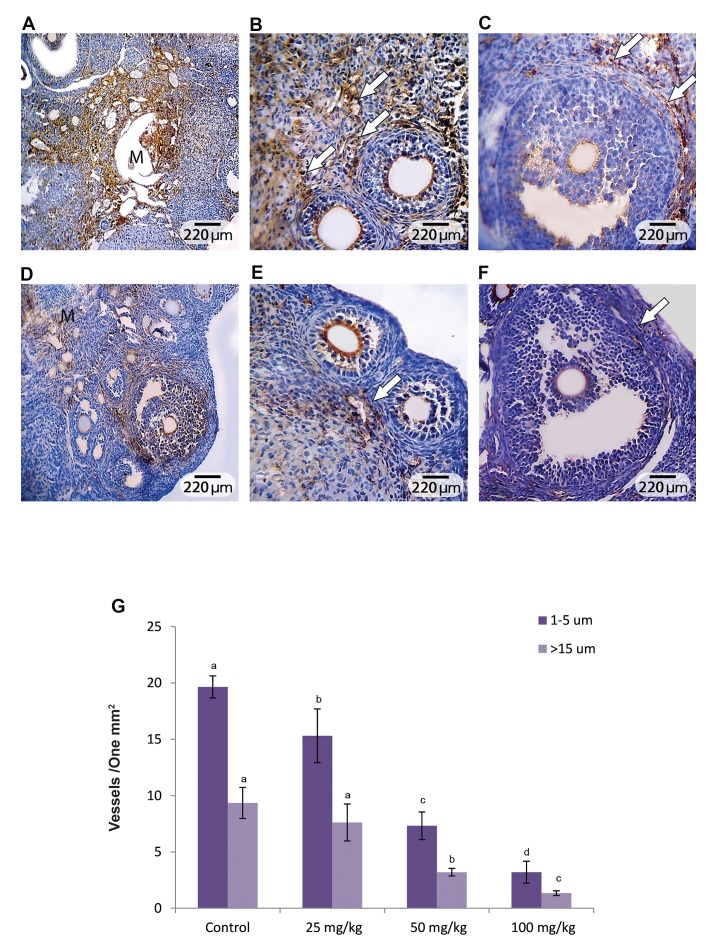
Immunohistochemical assessment of angiogenesis and vascular distribution. A. Control, B. The high dose pulegone (PGN)-exposed group. Cross section of the ovary from the PGN-exposed group represents a significant reduction in vascular distribution, C. Physiologic stromal vascularization is marked in the intact secondary follicles (arrows), D. Which is significantly decreased in cross a section from the PGN-exposed group, E. Angiogenesis in the theca layer of intact antral follicles, which are not developed in the theca layer (arrows) of F. Atretic antral follicles (Immunohistochemical staining for CD31, ×200 and ×400 magnifications), and G. Effect of PGN on mean average of 1-5 µm and 5-10 µm vessels distribution per one m of the ovarian tissue, all data are presented as mean ± SD. ^a, b, c, d^ represent significant differences (p<0.05) between groups.

The IHC staining for CD31 was performed and ovarian angiogenesis was estimated in all groups. Observations showed that PGN, in a dose dependent manner, reduced ovarian vascularization. The animals in the high dose (100 mg/kg) PGN group exhibited significantly (P<0.05) reduced vascular distribution per m of the ovarian cortex and medulla. The vasculature enclosed within the theca layer of the antral follicles significantly diminished in the PGN-exposed group. The stromal angiogenesis in the cortex region was investigated in order to evaluate the preantral follicles vascular support. The animals in PGN-receiving groups showed decreased vascular distribution per m of the cortical region and manifested reduced angiogenesis adjacent to the preantral follicles (Fig .4A-G).

### Pulegone altered expression of apoptosis relating genes

**Fig.5:**
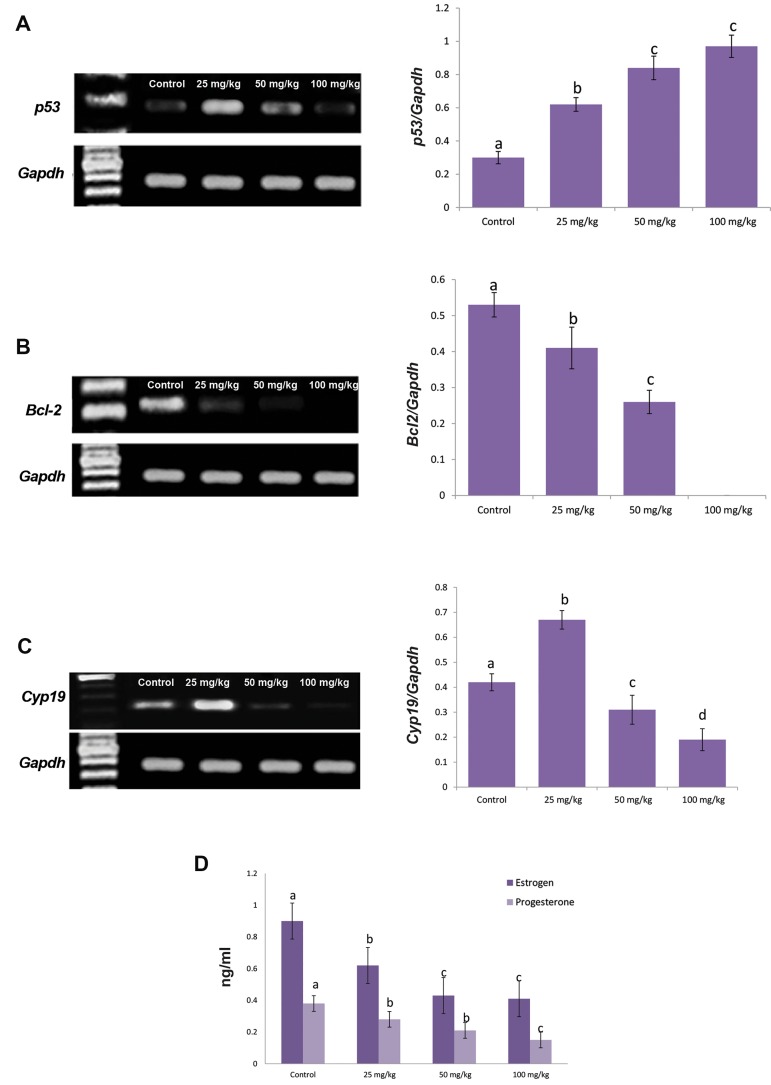
Effect of pulegone (PGN) on *p53, Bcl-2* and *Cyp19* mRNA levels in ovarian tissue as well as serum levels of estrogen and progesterone: the mRNA levels of A. *p53, B. Bcl-2, C. Cyp19* and *Gapdh* were evaluated by semi-quantitative reverse transcription polymerase chain reaction (RT-PCR), the density of mRNA bands for *p53, Bcl-2* and *Cyp19* was measured by densitometry and normalized to *Gapdh* mRNA expression level. Results were expressed as integrated density values (IDV) of *p53, Bcl-2* and *Cyp19* mRNA levels, and D. Effect of PGN on serum levels of estrogen and progesterone, all data are presented as mean ± SD. ^a,b,c,d^ represent significant differences (p<0.05) between groups.

The *p53* and *Bcl-2* mRNA levels were evaluated using RT-PCR analyses. The animals in the low (25 mg/kg) and medium (50 mg/kg) doses of PGN groups showed the highest *p53* mRNA levels, respectively. This situation was faint in the high dose (100 mg/ kg) PGN group. More analyses for *Bcl-2* exhibited a contrary pattern. The low (25 mg/kg) and medium (50 mg/kg) doses of PGN resulted in a significant (P<0.05) reduction in *Bcl-2* mRNA levels. No band for the mRNA of *Bcl-2* was revealed in the high dose (100 mg/kg) PGN group. However, the animals in the control group showed remarkably (P<0.05) higher *Bcl-2* and significantly (P<0.05) lower *p53* mRNAs in comparison to PGN-receiving animals (Fig .5A, B). 

### Pulegone reduced Cyp19 mRNA levels and affected serum concentrations of estrogen and progesterone

The RT-PCR analysis showed that PGN at doses of 25 mg/kg significantly (P<0.05) increased Cyp19 mRNA levels versus the control group. However, the mRNA levels of Cyp19 were significantly (p<0.05) decreased in medium (50 mg/kg) and high (100 mg/kg) doses of PGN groups (Fig .5C). Biochemical analysis showed that PGN, in a dose dependent manner, reduces the serum levels of estrogen and progesterone in comparison to control animals (Fig .5D).

### Pulegone affected ERα and ERβ expression

**Fig.6:**
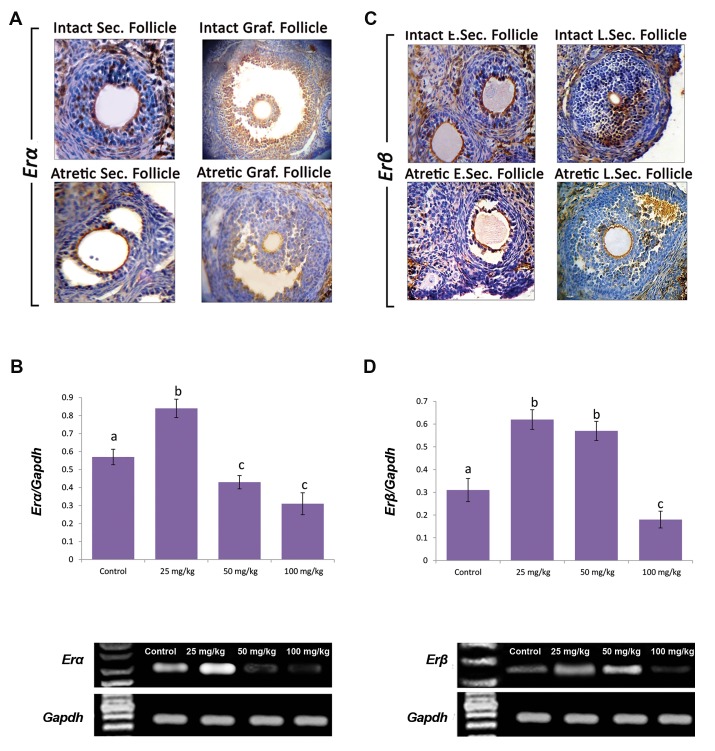
Effect of pulegone (PGN) on ERα and ERß proteins (see brown chromogen) and mRNA levels. A. Intact secondary (preantral) follicle with ERa expressionlevels in granulosa cells, which is significantly diminished in atretic secondary follicles. Normal graafian follicle with intensive ERα expression. Note reduceddistribution of ERα positive cells in atretic graafian follicles (×400 magnification), B. mRNA levels of *Erα* and *Gapdh* were evaluated by semi-quantitative reverse transcription polymerase chain reaction (RT-PCR), the *Erα* density measured by densitometry and normalized to *Gapdh* mRNA expression, C. Immunohistochemical staining for ERß: ERß expression in intact early secondary, late secondary follicles (preantral), which is significantly decreased in atreticearly and late secondary follicles, (×400 magnification), and D. Effect of pulegone (PGN) on mRNA levels of *Erß* in ovarian tissue: mRNA levels of *Erß* and *Gapdh* were evaluated using semi-quantitative RT-PCR. The Erß density measured by densitometry and normalized to Gapdh mRNA expression level. Results were expressed as integrated density values (IDV) of *Erß* mRNA level. ^a, b, c^ represent significant differences (p<0.05) between groups.

The ERα and ERβ proteins and the mRNA levels were estimated using IHC staining and RT-PCR analysis, respectively. Observations showed that, the mRNA level of ERα significantly (P<0.05) increased in the low dose (25 mg/kg) PGN-receiving animals versus those in the control, medium and high dose PGN groups. However, in a dose dependent manner, the mRNA level of ERα significantly (P<0.05) decreased in the medium (50 mg/kg) and high dose (100 mg/kg) groups. IHC staining demonstrated that the follicular cells of intact antral follicles and stromal cells of the ovaries (in control groups) exhibited ERα that was remarkably decreased in atretic follicles of the same stage (Fig .6A, B). More analyses for ERβ showed that PGN causes decreased expression of ERβ mRNA. Accordingly, the distribution of ERβ-positive cells was significantly (P<0.05) decreased in stromal cells enclosed to the preantral follicles. Moreover, the GCs and theca cells of antral follicles showed decreased ERβ protein compared to those in intact antral follicles (Fig .6C, D).

## Discussion

In the present study, we have analyzed IHC and RT-PCR expression data to measure the expression of *Erα* and *Erß* and defined that, PGN resulted in severe decrease in *Erα* and *Erß* expression at both the protein and mRNA levels. We have also established low levels of transcription for *Cyp19* in PGN-exposed animals. Expression was diminished in a dose dependent manner. Moreover, we found that elevated *p53* and decreased *Bcl-2* expression were both associated with enhanced follicular cell and oocyte apoptosis/necrosis and consequently follicular atresia. In order to clarify the exact mechanism(s) by which PGN negatively impacts the ovaries, special staining techniques were conducted to detect probable cases of induced apoptosis and/or necrosis. We showed that PGN more prominently at the low dose (25 mg/kg) and with lower efficiency at the medium dose (50 mg/ kg) triggered apoptosis that triggered necrosis at the high dose (100 mg/kg) of exposure. Finally, we found positive correlation between reduced *Cyp19* expression and reduced estrogen biosynthesis. 

The cytochrome P450 (P450) enzymes family consists of constitutive and inducible mono-oxygenase enzymes that metabolize many lipophilic, biologically active endogenous and xenobiotic substrates including, a large number of therapeutic drugs and toxic environmental chemicals (27-29). Actually, it has been reported that *Cyp19* oxidizes PGN to menthofuran (2). Considering the direct involvement of *Cyp19* in metabolizing PGN, we can come to the conclusion that PGN treatment resulted in compensatory expenditure of *Cyp19*, which in turn affected biosynthesis of estrogen from androgens. To uncover the relation and/or association between *Cyp19* and estrogen, it should be considered that in ovaries, the key genes that are involved in encoding the aromatase cytochrome P450 are co-expressed with estrogen, suggesting estrogen-induced paracrine or autocrine effects (30). Early studies showed that at the early and late preantral stages, follicles possess gonadotrophins that initiate estrogen synthesis (31). Accordingly, the preovulatory follicle has the highest intrafollicular levels of estradiol, primarily due to the size of its GCs population and its capacity for androgen aromatization. Although aromatase activity is present in small antral follicles, estrogen production at this stage of development is limited by an inability to produce the androgen substrate for aromatization to estrogen (32). The current study shows that PGN, in a dose dependent way, enhances follicular atresia and ovarian tissue necrosis. Therefore, it would be more logic to conclude that, both reduced Cyp19expression and the associated atresia of preovulatory follicles resulted in a remarkable reduction in estrogen biosynthesis. Dose dependent reduction of serum estrogen confirmed this hypothesis. On the other hand, it should be considered that estrogen signals act via two forms of estrogen receptors as ERα and ERß. Accordingly, previous reports showed that ER knockout mice (*ERKO*) have the most severe ovarian phenotype, in which follicles fail to mature or ovulate and form hemorrhagic cysts, leading to infertility (33). In low dose (25 mg/kg) PGN-exposed animals, the expression of *Erα* and *Erß* increased both at the protein and the mRNA levels, suggesting compensatory transcription/ biosynthesis of these receptors following a decrease in estrogen. However, this situation was inversed at higher doses (50 mg/kg and 100 mg/kg). Indeed, estrogen promotes proliferation of GCs, oocyte development and provokes follicles to escape from atresia and reach the preovulatory stage via *Erα* and *Erß* receptor signaling pathways (33, 34). Thus, reduced *ERs* expression associated with decreased aromatization potentially resulted in severe follicular atresia, suggesting a PGN-induced impact on vital interactions between aromatization, estrogen levels and *ER* signaling pathways. 

Over the last few years there has been increasing evidence that expression of certain genes, such as *p53* and *Bcl-2*, may affect the cellular response to an apoptotic stimulus (22, 35). There is an inverse relationship between *Bcl-2* and *p53*. Accordingly, after cells have been exposed to apoptotic stimuli, *p53* (36) and *Bcl-2* (22) are associated positively and negatively with release of cytochrome C from the mitochondria into the cytoplasm, respectively. Indeed, *p53* is known as a promoter for activating cell death proteases and *caspase III* expression (37). However, *Bcl-2* is considered as an apoptosis inhibitor protooncogene, which is involved in cell survival (22). 

Considering the point that GCs are the primary site for apoptosis during follicular atresia, we aimed to estimate the possible roles of *p53* and *Bcl-2* in PGN-exposed ovaries. The RT-PCR analysis showed that PGN up- regulated p53 expression and reduced *Bcl-2* expression at doses of 25 mg/kg and 50 mg/kg, suggesting an apoptotic effect of PGN at these doses. 

Histological investigations for apoptosis confirmed these alterations by revealing intensive apoptosis in low (25 mg/ kg) and medium dose (50 mg/kg) PGN-exposed ovaries. Meanwhile, we failed to measure *Bcl-2* mRNA in the high dose (100 mg/kg) PGN-exposed group but RT-PCR analysis illustrated decreased *p53* expression. Therefore, we considered the fact that PGN adversely affects ovarian follicular growth through other mechanism(s). Special fluorescent staining for mRNA damage in necrotic cells was performed to uncover possible necrotic impacts of PGN. Observations showed that, the ovaries from the group exposed to the high dose (100 mg/kg) of PGN exhibited severe necrosis, suggesting two different dose dependent effects of PGN on the female reproductive system.

Our IHC staining for studying angiogenesis revealed a significant reduction in ovarian angiogenesis in PGN- exposed animals. Previous observations suggest the potential for sex steroids to influence angiogenesis in ovarian tissues (38). Estrogen stimulates endothelial cell proliferation and migration in the ovarian tissue through ERs, which are expressed by the endothelial cells (39). 

In line with this issue, early studies showed that, estrogen induces the expression of vascular endothelial growth factor in ovaries and uterine tissue (18, 38). Thus, we can conclude that PGN adversely affects the ovarian angiogenesis progression by down-regulating estrogen biosynthesis associated with reduced *ERs* expression. More biochemical analyses showed that, PGN significantly diminished the serum levels of progesterone versus the control group. With that in mind, the ovulation and consequent corpora lutea formation positively correlate with serum levels of progesterone, it would be more logic to conclude that, the PGN-induced apoptosis and necrosis negatively affects the survival of graafian follicles, leading to lower ovulation and serum levels of progesterone compared to control animals.

## Conclusion

The PGN enhanced follicular atresia by multiple mechanisms including; i. Reducing aromatization, ii. Down-regulating estrogen synthesis, iii. Altering the expression of *Bcl-2* and *p53*, iv. Diminishing ERs (*Erα* and *Erß*) expression, and v. Reducing angiogenesis. 
